# Reproductive Neuroendocrine Pathways of Social Behavior

**DOI:** 10.3389/fendo.2016.00028

**Published:** 2016-03-31

**Authors:** Ishwar S. Parhar, Satoshi Ogawa, Takayoshi Ubuka

**Affiliations:** ^1^Brain Research Institute, School of Medicine and Health Sciences, Monash University Malaysia, Bandar Sunway, Malaysia

**Keywords:** RFamide peptides, aggression, depression, sex behaviors, anxiety

## Abstract

Social behaviors are key components of reproduction, because they are essential for successful fertilization. Social behaviors, such as courtship, mating, and aggression, are strongly associated with sex steroids, such as testosterone, estradiol, and progesterone. Secretion of sex steroids from the gonads is regulated by the hypothalamus–pituitary–gonadal (HPG) axis in vertebrates. Gonadotropin-releasing hormone (GnRH) is a pivotal hypothalamic neuropeptide that stimulates gonadotropin release from the pituitary. In recent years, the role of neuropeptides containing the C-terminal Arg–Phe–NH_2_ (RFamide peptides) has been emphasized in vertebrate reproduction. In particular, two key RFamide peptides, kisspeptin and gonadotropin-inhibitory hormone (GnIH), emerged as critical accelerator and suppressor of gonadotropin secretion. Kisspeptin stimulates GnRH release by directly acting on GnRH neurons, whereas GnIH inhibits gonadotropin release by inhibiting kisspeptin, GnRH neurons, or pituitary gonadotropes. These neuropeptides can regulate social behavior by regulating the HPG axis. However, distribution of neuronal fibers of GnRH, kisspeptin, and GnIH neurons is not limited within the hypothalamus, and the existence of extrahypothalamic neuronal fibers suggests direct control of social behavior within the brain. It has traditionally been shown that central administration of GnRH can stimulate female sexual behavior in rats. Recently, it was shown that Kiss1, one of the paralogs of kisspeptin peptide family, regulates fear responses in zebrafish and GnIH inhibits sociosexual behavior in birds. Here, we highlight recent findings regarding the role of GnRH, kisspeptin, and GnIH in the regulation of social behaviors in fish, birds, and mammals and discuss their importance in future biological and biomedical research.

## Introduction

Reproduction is an essential process in vertebrates, which consists of various aspects of physiological events throughout the lifespan, including fertilization, development, puberty, social and sexual behaviors, maturation, and aging. Reproductive functions are controlled by the hypothalamus–pituitary–gonadal (HPG) axis. The hypothalamus, a central brain region that is responsible for the control of reproduction, regulates pituitary hormone synthesis and release. Gonadotropin-releasing hormone (GnRH) or luteinizing hormone (LH)-releasing hormone is a pivotal hypothalamic neuropeptide that regulates vertebrate reproduction (1). In tetrapods, GnRH neurons are located in the preoptic–hypothalamic region and project to the median eminence to regulate gonadotropin synthesis and release from the anterior pituitary gland, which stimulates sex steroid secretion and gametogenesis. It was also classically shown that central administration of GnRH can stimulate female sexual behavior in rats (2, 3).

In recent years, the role of neuropeptides containing the C-terminal Arg–Phe–NH_2_ (RFamide peptides) has been emphasized in vertebrate reproduction. In particular, two key RFamide peptides: kisspeptin and gonadotropin-inhibitory hormone [(GnIH) also known as LPXRFamide peptides] emerged as critical regulators (accelerator and suppressor, respectively) of vertebrate reproduction. These neuropeptides have been identified in a variety of species, including non-mammalian vertebrates, and shown to have evolutionarily conserved functions (4). Although knowledge about the role of RFamide peptides in social behaviors is still limited, recent studies have shown that Kiss1, one of the paralogs of kisspeptin peptide family, regulates fear responses in zebrafish (5), and GnIH inhibits sociosexual behavior in birds (6–9).

Social behaviors are key components of reproductive functions, because they are essential for successful fertilization. As social behaviors, such as courtship, mating, and aggression, are strongly associated with sex steroids, such as testosterone, estradiol, and progesterone (10), hypothalamic neuropeptides can regulate social behaviors by regulating the HPG axis. However, neuronal fibers containing neuropeptides that regulate the HPG axis and their receptors are widely distributed outside of the hypothalamus, including limbic brain structures in the brain. Investigation of the neural mechanisms and functions of neuropeptides that regulate gonadotropin secretion in the regulation of social behavior has a potential to uncover fundamental regulatory mechanism of social behavior. Therefore, we highlight traditional and recent findings regarding the function of GnRH, kisspeptin, and GnIH neuropeptides in the regulation of social behaviors in fish, birds, and mammals and discuss their importance for further biological and biomedical researches in this article.

## Gonadotropin-Releasing Hormone

In the early 1970s, Schally’s and Guillemin’s groups independently reported the amino acid sequence of mammalian GnRH peptide that was extracted from pig and sheep hypothalami, respectively (11, 12) (Table 1). Orthologous peptides to mammalian GnRH, categorized as GnRH1, which have few substitutions in the amino acid sequence, have been identified in other vertebrates, such as guinea pig (13), chicken (14, 15), and sea bream (16) (Table 1). It was shown that the expression of GnRH1 precursor mRNA is developmentally and seasonally regulated in songbirds (17, 18). In addition to the hypothalamic GnRH1, there are non-hypothalamic types of GnRH (GnRH2 and GnRH3) and multiple GnRH receptors in most vertebrate species (19). GnRH2 is the most evolutionarily conserved form of GnRH, which many vertebrate species possess the identical peptide that was first identified in the chicken (20) (Table 1). GnRH2 neuronal cell bodies exist in the midbrain in all vertebrates investigated (21). GnRH3 was first identified in the salmon (22) (Table 1). GnRH3 neurons are present in the terminal nerve ganglion, and neuronal fibers were localized at the junction of the olfactory nerve and the telencephalon in most teleost species (23). As these extrahypothalamic GnRH neural populations project their neural fibers throughout the brain, their primary role may be to regulate social behavior by modulating other neurons in the brain. Indeed, in marmoset monkey, musk shrew, and white-crowned sparrows, GnRH2 enhances female reproductive behavior (24–27). In goldfish, both GnRH2 and GnRH3 significantly stimulate female spawning behavior (28). In cichlid fish, terminal nerve GnRH3 neurons regulate male social behaviors, including nest building and territorial behaviors (29). A recent study in Japanese medaka revealed a novel function of terminal nerve GnRH3 neurons as a gate for activating mating preferences based on familiarity (30).

**Table 1:** **Representative amino acid sequences of GnRH, kisspeptin, and GnIH peptide families in mammals, birds, and teleost fishes**.

Vertebrates	Peptide family	Peptide name	Amino acid sequence	Reference
Mammals	GnRH	Mammalian GnRH	pQHWSYGLRPGamide	Matsuo et al. (11) and Burgus et al. (12)
		Guinea pig GnRH	pQYWSYGVRPGamide	Jimenez-Liñan et al. (13)
	Kisspeptin	Human KISS	YNWNSFGLRFamide	Lee et al. (31)
		Mouse Kiss	YNWNSFGLRYamide	Stafford et al. (32)
	GnIH	Human RFRP1	MPHSFANLPLRFamide	Ubuka et al. (33)
		Human RFRP3	VPNLPQRFamide	Ubuka et al. (33)
Birds	GnRH	Chicken GnRH1	pQHWSYGLQPGamide	King and Millar (14) and Miyamoto et al. (15)
		Chicken GnRH2	pQHWSHGWYPGamide	Miyamoto et al. (20)
	GnIH	Quail GnIH	SIKPSAYLPLRFamide	Tsutsui et al. (34)
		Quail GnIH-RP2	SSIQSSLLNLPQRFamide	Satake et al. (35)
Teleost fishes	GnRH	Sea bream GnRH1	pQHWSYGLSPGamide	Powell et al. (16)
		Salmon GnRH3	pQHWSYGWLPGamide	Sherwood et al. (22)
	Kisspeptin	Zebrafish Kiss1	YNLNSFGLRYamide	Biran et al. (36)
		Zebrafish Kiss2	FNYNPFGLRFamide	Kitahashi et al. (37)
	GnIH	Goldfish LPXRFa3	SGTGLSATLPQRFamide	Sawada et al. (38)

Traditionally, hypothalamic GnRH (GnRH1) has also been shown to regulate reproductive behaviors, including female lordosis (39) and male mating behavior (40) in rats. In addition to sexual behaviors, hypothalamic GnRH is also known to modulate other social behaviors. In rhesus monkeys, treatment with a GnRH-receptor antagonist Antide, during neonatal periods, alters their social behaviors, such as group in proximity and grooming behaviors (41). In various mammalian species, immunization or immunoneutralization against GnRH results in reduction of aggressiveness (42–44). These results, however, have traditionally been thought to be mainly due to reduction of gonadal hormone release and not due to reduced action of GnRH in the brain, as relatively longer treatment was required. However, accumulating evidences suggest possible direct action of GnRH1 within the brain as a neurotransmitter or a neuromodulator, because GnRH1 receptor is expressed outside of hypothalamus and pituitary (45–47). In male hamster, GnRH1 enhances the main olfactory input to the medial amygdala, which may be important for receiving conspecific reproductive chemosignals (48).

In addition to reproductive functions, GnRH is also associated with anxiety and mood disorders, such as depression, because adverse effects of GnRH agonists have been observed in women undergoing assisted reproductive treatment (49, 50). In rodent models, GnRH agonists exhibit anxiolytic- and antidepressant-like effects, whereas GnRH antagonists induce anxiogenic-like behavior (51, 52), although the neuronal mechanism underlying the role for GnRH in mediating anxiety and depression has not been understood well. One possibility is that GnRH may regulate other neuropeptides that mediate emotional behaviors and stress responses (53). Interactions between vasopressin, a stress hormone that mediates social- and anxiety-like behaviors, neurons and GnRH neurons have been observed in the supraoptic nucleus of monkeys (54). In rats, GnRH agonist stimulates the release of vasopressin from the neurohypophysis (55). It was shown that GnRH2 inhibits food intake (56), and the anorexigenic action of GnRH2 neuron is regulated by various neuropeptides, including α-melanocyte-stimulating hormone and corticotropin-releasing hormone in goldfish (57).

## Kisspeptin

Kisspeptin is a family of peptides encoded by the KISS1 gene, which includes metastin (kisspeptin-54) and kisspeptin-10 (4). Comparison of amino acid sequences of kisspeptin among vertebrate species shows that the C-terminal 10 amino acid sequence is highly conserved, suggesting the importance of the core 10 amino acid region (4) (Table 1). The shortest endogenous 10 amino acid kisspeptin exerts equal receptor (GPR54)-binding activity as the other longer endogenous fragments (58, 59). In teleost fish, two forms of kisspeptin (Kiss1 and Kiss2) have been reported (37, 60) (Table 1). On the other hand, birds do not possess either kisspeptin or GPR54 gene (61).

Kisspeptin and its cognate receptor GPR54-signaling were reported to be involved in the stimulatory regulation of GnRH neurons (62–65). However, there are no defects in gender-specific sexual behaviors in GPR54-knockout mice as long as the appropriate sex steroid hormones are provided (66). Similarly, double-kisspeptin (kiss1 and kiss2) and kisspeptin receptors (kissr1 and kissr2) gene mutant lines are capable of achieving successful reproduction in zebrafish (67). These observations suggest that the central kisspeptin–GPR54 system is not essential for direct regulation of sexual behaviors.

Recently, we have identified *Kiss1* gene expressed in the ventral habenula (vHb) in the modulation of serotonin (5-HT) neurons and fear responses in the zebrafish (5, 68). Expression of *Kiss1* gene was also shown in the medial amygdala, a fear-regulating region in rodents (69). Furthermore, central administration of kisspeptin-13 increased basal corticosterone levels and induced hyperthermia upregulating motor behavior, causing anxiety in rats (70). In mice, kisspeptin-13 showed antidepressant-like effects in a modified forced swimming test *via* adrenergic and serotonergic receptors (71). It has also been shown that kisspeptin-13 facilitates learning and memory consolidation in a passive avoidance paradigm *via* various neurotransmitters in mice (72). Our very recent findings in the zebrafish suggest the interaction between the vHb-expressing Kiss1 and the 5-HT system in the modulation of alarm substance-evoked fear responses mediated *via* two serotonin receptor subtypes (73). These results suggest that kisspeptin can act on several brain regions to facilitate a variety of social behaviors *via* interaction with different types of neurotransmitters.

## Gonadotropin-Inhibitory Hormone

**Figure 1:**
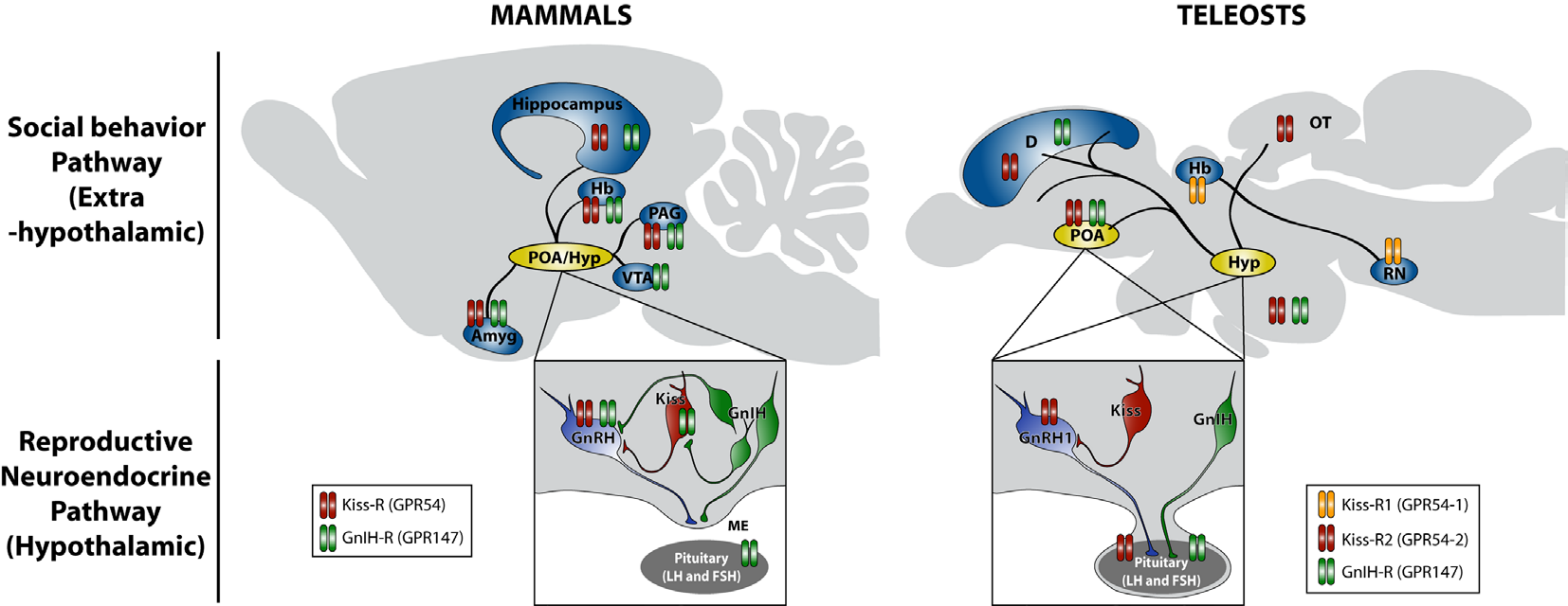
**Schematic model of actions of kisspeptin and GnIH in the regulation of social behavior in mammals and teleosts**. Neuronal cell bodies producing gonadotropin-releasing hormone (GnRH), kisspeptin (Kiss), and gonadotropin-inhibitory hormone (GnIH) are located in the preoptic area (POA) and hypothalamic (Hyp) region. GnRH is secreted at the median eminence (ME) in mammals, whereas GnRH1 is directly secreted in the pituitary in teleosts, and they regulate gonadotropin (LH and FSH) synthesis and release from the pituitary gland, which stimulates sex steroid synthesis and gametogenesis in the gonads. Sex steroids feedback to the brain and construct neuronal architecture and modulate the activity of neurons, which regulate the expression of social behavior, such as courtship, mating, and aggression. Kiss neurons stimulate GnRH and GnRH1 release in mammals and teleosts, respectively. GnIH neurons inhibit the activity of GnRH and Kiss neurons as well as pituitary gonadotropin secretion in mammals. On the other hand, GnIH neurons terminate in the pituitary in teleosts. In addition to this reproductive neuroendocrine (hypothalamic) pathway, neuronal fibers containing Kiss and GnIH are found in extrahypothalamic regions, such as amygdala (Amyg), hippocampus, habenula (Hb), periaqueductal gray (PAG), and ventral tegmental area (VTA) in mammals and also dorsal telencephalic area (D), optic tectum (OT), and raphe nuclei (RN) in teleosts, which can directly regulate social behavior by acting within the brain (social behavior pathway). Neuronal fiber distributions of Kiss and GnIH neurons as well as locations of kisspeptin receptor (Kiss-R: GPR54) and GnIH receptor (GnIH-R: GPR147) in mammals are based on Tena-Sempere (84), Lehman et al. (85), Tsutsui and Ubuka (86), and Ubuka et al. (74). Neuronal fiber distributions of Kiss and GnIH neurons as well as locations of kisspeptin receptor (Kiss-R1: GPR54-1 and Kiss-R2: GPR54-2) and GnIH receptor (GnIH-R: GPR147) in teleosts are based on Escobar et al. (87), Qi et al. (88), Nathan et al. (73), Parhar et al. (89), and Grone et al. (90).

Gonadotropin-inhibitory hormone has been discovered as a novel hypothalamic RFamide peptide that inhibits LH release in birds (34, 74). GnIH is also named RFamide-related peptide (RFRP) in mammals (75). GnIH orthologous peptides have characteristic LPXRFamide (X = L or Q) amino acid sequence at their C-termini. Endogenous GnIH peptides were identified in humans (33), quail (34, 35), goldfish (38), and in other vertebrates (74) (Table 1). The presence of orthologous GnIH receptor (GPR147) has also been demonstrated in various vertebrate species, suggesting that the GnIH–GPR147 signaling is evolutionarily conserved (76, 77). GnIH neurons terminate on GnRH neurons as well as kisspeptin neurons and these neurons express GPR147 (74, 78, 79) (Figure 1). In addition, GnIH–GPR147 signaling is regulated by various factors, such as natural and social environmental cues (79–81) and stress (82, 83), suggesting that GnIH is one of the mediators of favorable and unfavorable external stimuli (4). GnIH and GPR147 have been cloned and localized, and their functions have also been studied in several teleost species. However, the role of fish GnIH–GPR147 signaling remains inconclusive, because the physiological properties of fish LPXRFa are variable depending on reproductive condition and season.

Because GnIH neurons terminate in the close proximity of GnRH2 neurons (78, 91) and GnRH2 neurons express GPR147 (78), GnIH may inhibit reproductive behavior by inhibiting GnRH2 neuronal activity. In line with this hypothesis, Bentley et al. (92) showed that centrally administered GnIH inhibits copulation solicitation in estrogen-primed female white-crowned sparrows exposed to the song of males. Ubuka et al. (6) investigated the effect of RNA interference (RNAi) of the GnIH gene on the behavior of male and female white-crowned sparrows. GnIH RNAi reduced resting time, spontaneous production of complex vocalizations, and stimulated brief agonistic vocalizations. GnIH RNAi further enhanced song production in male birds when they were challenged by playbacks of novel male songs. Because these behaviors resembled behavior of breeding birds during territorial defense, it was suggested that GnIH gene silencing induces arousal (6). It was recently shown that GnIH directly activates aromatase neurons in the preoptic area and increases neuroestrogen synthesis beyond its optimum concentration for the expression of sociosexual behavior of male birds (8). Johnson et al. (93) showed that central administration of RFRP-3 significantly suppresses all facets of male sex behavior in rats. Central administration of GnIH reduced sexual motivation and vaginal scent marking, but not lordosis behavior in female hamsters (94). On the contrary, there was no effect of GnIH on sexual behavior in non-human primates and ewes (95), which could be due to different injection conditions or social or reproductive status of the animals used.

## Regulation of Reproductive Neuroendocrine Pathway by Social Interaction

Social interactions have significant effects on reproductive physiology and behavior in vertebrates (7, 96, 97). Male courtship behavior can greatly enhance the reproductive activity of female birds (98). Maney et al. (99) investigated the effect of male song on the rapid changes in LH and the induction of the immediate early gene Egr-1 in GnRH1 neurons in female white-throated sparrows. However, although male song induced LH release, it did not alter Egr-1 expression in GnRH1 neurons (99). Calisi et al. (100) manipulated nesting opportunities for pairs of songbirds and measured GnIH mRNA and GnIH content, as well as GnRH1 content and plasma testosterone concentration. The birds with nest boxes had significantly fewer numbers of GnIH cells than those without nest boxes, whereas GnRH1 content and testosterone concentration did not vary with nest box ownership, suggesting that GnIH may modulate reproductive behaviors without changing the HPG axis in response to social environment (100).

Olfactory cues significantly impact sexual attraction and behavior in mammals (101). The chemosignals that act between members of the same species and triggering short-term behavioral responses or long-term physiological changes are termed pheromones (102, 103). When prepubertal females are exposed to pheromones of sexually mature males, puberty onset is accelerated in rodents (104). Another example of pheromonal stimulation of reproductive activity is the “male effect” in domestic ungulates, sheep, and goat (103). If anestrus females are exposed to a male, their HPG axis will be reactivated leading to ovulation. De Bond et al. (105) showed that male introduction leads to elevated LH pulse amplitude and frequency in a non-breeding female. However, central infusion of kisspeptin antagonist in advance abolished the effect of male exposure on LH secretion, suggesting that the “male effect” is mediated by kisspeptin signaling in ewes (105). Murata et al. (106) showed that brief exposure of male pheromone induces multiple-unit activity at close proximity to kisspeptin neurons in the goat arcuate nucleus, a brain region that is thought to be the site of GnRH pulse generator (107).

## Clinical Perspectives of Neuropeptides Regulating Reproduction in the Treatment of Mood, Pain, or Stress-Related Disorders

During reproductive aging and reproductive cycles, plasma steroid levels alter considerably and cause significant influences on various aspects of physiological functions, including mental and cognitive functions. For example, it is well known that many women have fluctuations in mood and libido in conjunction with phases of the menstrual cycle. Accordingly, failure in homeostatic control of the HPG axis leads to disorders in mood and libido. Neuropeptides and their receptors have been recognized as therapeutic targets for various mental disorders, such as mood, depression, and anxiety (53, 108, 109). Recently, RFamide peptides have been recognized as new therapeutic targets (110, 111). Kisspeptin has recently been utilized for treatment of women with reproductive dysfunctions, although there are still very limited clinical cases (112–115). It was reported that citalopram, a potent selective serotonin reuptake inhibitor that is used as an antidepressant but causes sexual dysfunction, induced inhibition of sexual behavior involves stimulation of GnIH neurons through serotonin receptors in the rat (116), suggesting the use of GnIH receptor antagonist in the treatment of sexual dysfunction.

It is thought that GnIH gene and NPFF, a neuropeptide that has a PQRFamide motif at its C-terminal and involved in pain modulation, gene have diverged from a common ancestral gene through gene duplication (117, 118). It is also thought that GPR147 and GPR74, NPFF receptor, are paralogous (76, 119). Mammalian RFamide peptides, GnIH (RFRP-1 and -3), neuropeptides AF and FF, prolactin-releasing peptides, kisspeptin, and QRFP/26RFa peptides are considered endogenous ligands for NPFF1 (GPR147), NPFF2 (GPR74), GPR10, GPR54, and GPR103, respectively (74). Elhabazi et al. (120) showed that all RFamide peptides efficiently activate GPR147 and GPR74. As NPFF modulates morphine analgesia (121, 122), the hyperalgesic and anti-morphine-induced analgesic effects of endogenous RFamide peptides were analyzed in mice. All of the peptides induced hyperalgesia and/or prevented morphine analgesia following the central administration. These results show that all endogenous RFamide peptides display pain-modulating properties and that GPR147 and GPR74 are essential players for these effects (120), suggesting potential use of RFamide peptides, namely, GnIH and kisspeptin, for the treatment of pain and stress-related disorders.

## Summary and Conclusion

A variety of hypothalamic neuropeptides have been also identified as important regulators of social behaviors as neurotransmitter or neuromodulator (123). Expression of hypothalamic neuropeptides or activity of hypothalamic neurons also changes profoundly according to social environment to increase reproductive fitness (97). Social behaviors, such as affiliation, communication, and aggression, are closely associated with reproductive functions to ultimately achieve successful reproduction. We highlighted classical and recent findings regarding the role of GnRH, kisspeptin and GnIH, neuropeptides that are involved in gonadotropin secretion and in the regulation of social behaviors in fish, birds, and mammals and discussed their importance in future researches. The accumulating results suggest that these neuropeptides may directly regulate social behaviors by acting within the brain, besides regulating the HPG axis.

## Author Contributions

All authors listed have made substantial, direct, and intellectual contribution to the work and approved it for publication.

## Conflict of Interest Statement

The authors declare that the research was conducted in the absence of any commercial or financial relationships that could be construed as a potential conflict of interest.
